# Análisis del impacto presupuestal en Colombia de la prueba de HPV con genotipificación comparada con la citología

**DOI:** 10.7705/biomedica.6016

**Published:** 2022-06-01

**Authors:** Miguel Amézquita, Geny Carolina Silva, Diego Antonio Restrepo, Linda Margarita Ibata, Rafael Niño, Maximiliano Bustacara, Víctor Alexander Sáenz, Dieric Anderson Díaz, Milena Alarcón, Luz Adriana Quintero

**Affiliations:** 1 Economía de la Salud, InValue Health Solutions S.A.S., Bogotá, D.C., Colombia InValue Health Solutions S.A.S. Bogotá, D.C. Colombia; 2 Unidad Salud, Compensar EPS, Bogotá, D.C., Colombia Compensar EPS Bogotá, D.C. Colombia

**Keywords:** costos de la atención en salud, neoplasias del cuello uterino, infecciones por papilomavirus, Papillomaviridae, técnicas citológicas, tamizaje masivo, Health care costs, uterine cervical neoplasms, papillomavirus infections, Papillomaviridae, cytological techniques, mass screening

## Abstract

**Introducción*.*:**

La detección del virus del papiloma humano mediante la combinación de la prueba de HPV y otras técnicas como la citología, ha demostrado su eficacia en el diagnóstico y tratamiento oportuno de lesiones asociadas con el cáncer de cuello uterino.

**Objetivo.:**

Estimar el impacto presupuestal de la estrategia de detección temprana del HPV mediante la prueba de genotipificación combinada con la citología en comparación con la citología convencional, en mujeres de 30 a 65 años participantes en el programa de tamizaje de cáncer de cuello uterino en una Entidad Administradora del Plan de Beneficios en salud (EAPB) en Colombia.

**Materiales y métodos.:**

Utilizando un árbol de decisiones y un modelo de Markov, se estimaron las implicaciones clínicas y los costos directos anuales de dos ciclos de tamizaje, diagnóstico y tratamiento, en una cohorte de mujeres. Las prevalencias de los resultados clínicos y los costos se tomaron de la base de datos de una EAPB y la información de la progresión, persistencia y regresión de los estados de salud provinieron del estudio ATHENA.

**Resultados.:**

El esquema de tamizaje con la prueba de HPV, la genotipificación y la citología resultó en un ahorro de costos comparado con la citología convencional. El costo promedio por ciclo de tamizaje con la prueba de HPV se estimó en COP $129’201.363 y con la citología en COP $186’309.952, es decir, un ahorro de COP $57’108.589 (30,7 %).

**Conclusión.:**

La implementación de la estrategia de tamizaje evaluada sugiere que habría ahorros derivados de la detección temprana de los estados de salud asociados con el desarrollo de cáncer de cuello uterino.

## Abstract (en)

**Introduction:**

The detection of the human papillomavirus (HPV) through the combination of the HPV test and other techniques such as cytology has impacted the detection and timely treatment of lesions associated with cervical cancer.

**Objective:**

To estimate the budgetary impact of the strategy of early detection of HPV with DNA test genotyping with reflex cytology versus conventional cytology in women aged 30 to 65 years attending the cervical cancer screening program at a health benefit managing entity in Colombia.

**Materials and methods:**

Using a decision tree and a Markov model, the clinical implications and direct costs of screening, diagnosis, and treatment were estimated in a cohort of women. The analysis considered two screening cycles and their annual costs. The data on the prevalence of clinical results and the costs were taken from the health managing entity. The information on the progression, persistence, and regression of the health states were taken from the ATHENA study.

**Results:**

The screening scheme with the HPV test, genotyping, and reflex cytology compared to conventional cytology was cost-saving. The average cost per screening cycle with the HPV test was estimated at COP $ 129,201,363 and with cytology at COP $ 186,309,952, i.e., a saving of COP $ 57,108,589 (30.7%).

**Conclusion:**

The implementation of the screening strategy under evaluation suggests prospective savings derived from the early detection of health states associated with the development of cervical cancer.

## Introducción

El cáncer de cuello uterino se origina en las células que revisten el cuello uterino. La infección persistente por el virus del papiloma humano (*Human Papilloma Virus*, HPV) es el factor de riesgo más importante en la mayoría de los casos ^1^. Los HPV son un grupo de más de 150 virus relacionados y a cada tipo se le ha asignado un número. Los tipos HPV-AR (alto riesgo) 16 y 18 son los causantes del 70 % del cáncer de cuello uterino y las lesiones precancerosas del cuello del útero, siendo el HPV 16 el de mayor riesgo para desarrollar cáncer *in situ* y cáncer invasor ^2^.

En el 2018, el cáncer de cuello uterino fue la tercera causa más frecuente de cáncer en mujeres a nivel mundial, y cada año se detectan más de medio millón de casos nuevos ^3^. En el 2017, en Colombia, este tipo de cáncer ocupó el segundo lugar entre las mujeres y el tercero en la población general ^4^, siendo la primera causa de muerte por cáncer entre mujeres de 30 a 59 años ^2^. Actualmente se estima que en el país mueren anualmente cerca de 2.000 mujeres por esta enfermedad, con una tasa de mortalidad de 5,7 por 100.000 pacientes ^5^. Por ello, la detección temprana del cáncer cervicouterino busca disminuir su incidencia en mujeres con riesgo de posibles lesiones premalignas, es decir, aquellas con signos de cáncer o cambios que puedan resultar en cáncer ^6^. Además, la detección temprana logra equilibrar la alta sensibilidad y el valor predictivo negativo con la alta especificidad de las pruebas de tamizaje, evitando, así, intervenciones ^7^.

Entre las pruebas de detección de ADN, la prueba Cobas™ ha demostrado una sensibilidad y especificidad óptimas, pues permite identificar genotipos de alto riesgo como los HPV-AR de tipos 16 y 18, y otros 12 serotipos asociados con el desarrollo de cáncer de cuello uterino. La detección de una carga viral elevada orienta el manejo oportuno de las pacientes con riesgo de aparición de carcinoma *in situ* antes de las alteraciones que puedan hallarse con la citología cervicovaginal ^8^^,^^9^, disminuyendo así la carga para el sistema de salud e impactando positivamente a las mujeres en términos de calidad de vida.

El objetivo del estudio fue estimar el impacto presupuestal de la implementación de la estrategia de detección temprana del virus del papiloma humano (HPV) con la prueba de genotipificación (prueba para HPV - Cobas™) y citología refleja, comparada con la citología convencional en mujeres de 30 a 65 años que asistieron al programa de tamizaje de cáncer de cuello uterino en una unidad de servicios de salud de Compensar EPS entre 2015 y 2018.

## Materiales y métodos

### Población de estudio

El modelo se aplicó en una cohorte de 10.219 mujeres de 30 a 65 años (rango de edad con una alta prevalencia de la infección por HPV) ^1^, examinadas en el marco del tamizaje primario del cáncer de cuello uterino en una unidad de servicios de salud de Entidad Promotora de Salud Compensar EPS. La misma cohorte se utilizó para estimar y comparar los dos escenarios de estudio sin estratificar según la edad. En la práctica habitual no todas las pacientes asisten a las citas de reevaluación, o no se presentan para la siguiente visita en el ciclo de detección, por lo que no cumplen con la frecuencia propuesta para cada una de las estrategias. Por ello, se estimó el porcentaje de mujeres que asistió a su segundo ciclo de tamizaje en la cohorte de la unidad de servicios de salud y se asumió el 4,28 % como el porcentaje de asistencia al segundo ciclo de tamizaje de la cohorte analizada.

### Opciones de tratamiento

Con el programa de prevención del cáncer de cuello uterino se busca detectar lesiones precancerosas de cuello uterino o carcinomas infiltrantes de cuello uterino en estadios tempranos, según la edad de la mujer ^1^.

En Colombia, el esquema habitual de tamizaje para mujeres de 30 a 65 años se hace con la citología de cuello uterino, la cual consiste en la detección de células anormales en el cuello uterino que pueden ser precancerosas o cancerosas, pero que también pueden deberse a otro padecimiento ^10^. Las células se obtienen mediante un cepillado o raspado ligero del cuello uterino y posteriormente se envían al laboratorio para su examen bajo el microscopio ^1^. A pesar de tener una sensibilidad entre baja y moderada, la citología ha permitido reducir la incidencia de cáncer de cuello uterino y la mortalidad en un 80 % en los países de mayores ingresos ^2^.

Sin embargo, en países de ingresos medios y bajos este tipo de cáncer continúa siendo un problema de salud pública, ya que aparte de la disponibilidad de las pruebas de detección de HPV, la disminución de la incidencia y la mortalidad por cáncer de cuello uterino se atribuyen a medidas y factores adicionales, como el nivel socioeconómico y la disminución de la infección por HPV-AR, y como resultado de mejoras en la higiene genital y en la disminución de la transmisión de enfermedades de transmisión sexual ^3^.

La prueba Cobas 4800™ (Roche Molecular Diagnostics, Pleasanton, CA, USA) es la única prueba clínicamente validada y aprobada por la *Food and Drug Administration* (FDA) que suministra simultáneamente resultados agrupados sobre los genotipos de alto riesgo y resultados individuales sobre los de mayor riesgo (HPV-AR). La prueba de detección de ADN es un sistema automatizado de PCR en tiempo real con iniciadores de la región L1 del HPV. Permite detectar directamente el virus de HPV en las células del cuello uterino y entre los resultados están los siguientes: el genotipo 16, el genotipo 18 y otros 12 genotipos de alto riesgo considerados patógenos o de alto riesgo para la enfermedad maligna del cuello uterino: 31, 33, 35, 39, 45, 51, 52, 56, 58, 59, 68, 66 ^11^. La prueba puede hacerse simultáneamente con la citología y con la misma escobilla algodonada u otra adicional ^1^.

### Modelo conceptual

**Figura 1:**
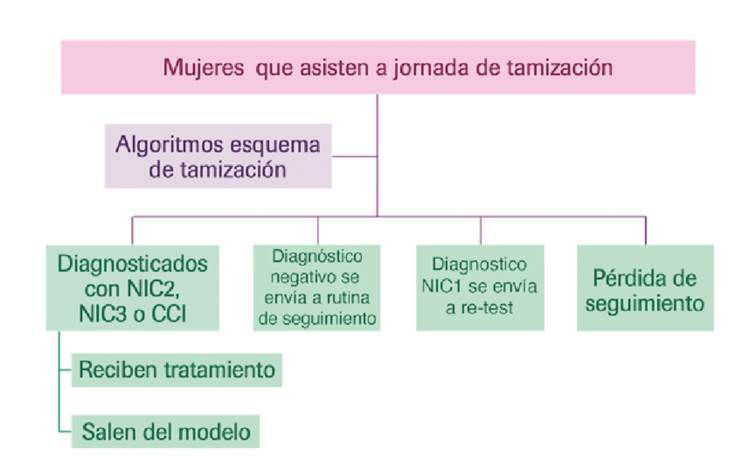
Árbol de decisión de la jornada de tamizaje

El análisis del impacto presupuestal parte de un árbol de decisiones basado en el desarrollo de los esquemas de tamizaje (figura 1). En este árbol se definen las posibles acciones en la práctica clínica según el resultado obtenido en las dos pruebas de tamizaje analizadas.

En el modelo propuesto se plantean los siguientes cuatro escenarios definidos según el resultado clínico que obtengan las mujeres en la jornada de tamizaje: (i) en los casos en que se diagnostiquen y confirmen mediante pruebas los estados de neoplasia intraepitelial de cuello uterino de grado 2+ (NIC2), neoplasia intraepitelial de cuello uterino de grado 3+ (NIC3 ) o cáncer invasivo de cuello uterino , las pacientes recibirán el tratamiento correspondiente y saldrán del modelo; (ii) aquellas con resultado negativo continuarán en la rutina de seguimiento, y (iii) cuando se detecta una NIC1, se hace una nueva prueba con citología refleja; por último, (iv) aquellas mujeres que no siguen los lineamientos de la tamizaje se asumen como pérdida en el seguimiento.

El análisis incluye también un modelo de Markov que reúne los estados clínicos de progresión y regresión para simular la historia natural de la enfermedad asociada con los estados neoplásicos (NIC1, NIC2, NIC3, CCI) por infección de HPV, lo que permite estimar los costos a largo plazo y los resultados en salud relacionados con la enfermedad de interés y las tecnologías de salud bajo estudio ^12^.

El modelo de Márkov tiene siete estados de salud: negativo para HPV, positivo para HPV, NIC1, NIC2, NIC3, CCI y muerte; la progresión se inicia con la identificación positiva de HPV en la prueba y puede avanzar a las neoplasias intraepiteliales de cuello uterino de diferentes grados (NIC1, NIC2, NIC3); a partir del estado NIC3 se progresa al cáncer invasivo de cuello uterino y de este a la muerte.

La posibilidad de regresión solo es posible en cinco de los estados del modelo y se explica por la oportuna intervención en las pacientes que no han desarrollado cáncer invasivo de cuello uterino: de NIC3 a NIC1 y negativas para HPV, de NIC2 a NIC1 y negativas para HPV, de NIC1 a positivas o negativas para HPV y, por último, de positivas a negativas para HPV. Lo anterior se basa en el modelo descrito por Wright, *et al.* en el análisis del impacto presupuestal de la detección del cáncer de cuello uterino mediante la detección primaria del HPV en Estados Unidos ^13^.

Asimismo, en el modelo se asumió como constante la posibilidad de progresión o regresión en el tiempo y no se estratificó según la genotipificación del virus. Además, no se consideró el impacto de la vacuna contra el HPV, ya que esta se incluyó en el Programa Ampliado de Inmunizaciones (PAI) de Colombia en el 2012 para niñas de 9 a 17 años ^3^. En el modelo no se diferenciaron las etapas del cáncer de cuello uterino, ni se incorporaron las mujeres con resultados de HPV-AR indetectables.

Por otra parte, se asumió que el total de las pacientes con NIC2, NIC3 y CCI fueron tratadas, por lo que se incurrió en costos de tratamiento y salieron del modelo de análisis, y, en consecuencia, no se incluyó la prueba de seguimiento al tratamiento ni el monitoreo. Las pacientes con NIC1 retomaron la detección de rutina o se enviaron a una nueva prueba en los tiempos definidos para cada una de las estrategias. Los resultados positivos para HPV-AR16/18 se enviaron directamente a prueba confirmatoria y si la colposcopia era negativa, se enviaba a una nueva genotipificación en 18 meses y no a la detección de rutina.

El desarrollo y análisis de los datos del árbol de decisión y del modelo de Markov estuvieron a cargo del equipo del Laboratorio Roche utilizando el programa de Microsoft Excel™ adaptado al contexto colombiano para evaluar el impacto en costos y resultados clínicos de los diferentes escenarios, incluidas las estrategias de detección durante dos ciclos de tamizaje rutinaria en la práctica clínica local.

### Escenarios

**Figura 2:**
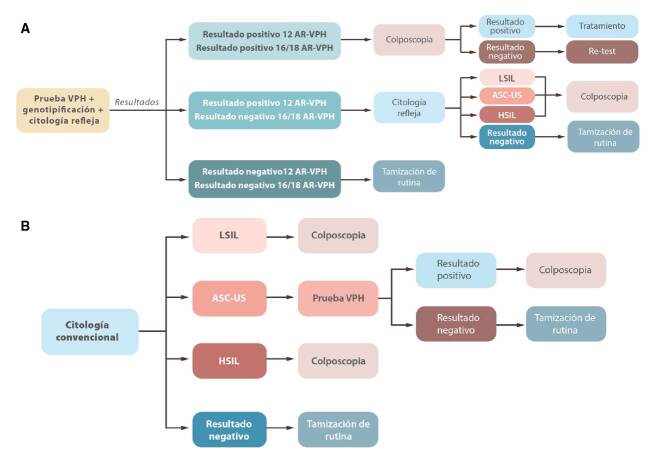
Esquemas de tamizaje de intervención y de comparación. a. Intervención: prueba de VPH con genotipificación y citología refleja. b. De comparación: citología convencional

Se desarrolló un escenario para la estrategia de tamizaje con citología siguiendo el esquema 1-1-3 (figura 2a), el cual consiste en una citología cada año durante dos años consecutivos, y ampliación del intervalo a tres años si el resultado de las dos es negativo ^2^. Sin embargo, el modelo solo tuvo en cuenta una única referencia de frecuencia de asistencia al tamizaje, por lo que a partir del esquema descrito, se asumió que las mujeres asistían a sus jornadas de citología a los 1,67 años, con un intervalo hasta la repetición de la prueba de seis meses.

La información proveniente de la vida real utilizada para este escenario se obtuvo de una de las unidades de servicios de salud de Compensar EPS que actualmente hace el tamizaje con citología convencional. Se planteó otro escenario con la estrategia de tamizaje mediante la prueba HPV y las siguientes consideraciones: si la prueba de HPV resultaba positiva y la citología refleja, negativa, se citaba nuevamente a un control o a una nueva prueba de HPV en 18 meses; si la prueba de HPV y la citología resultaban positivas, la paciente se remitía a colposcopia y biopsia según los hallazgos y, por último, si resultaba negativa, se contemplaba nuevamente el análisis a los cinco años (figura 2b). La información proveniente de la vida real para este escenario se obtuvo de otra de las unidades de servicios de salud de Compensar EPS en la que el tamizaje se hace con la prueba de HPV desde el 2015.

### Parámetros del modelo

*Datos clínicos*. La prevalencia de los resultados clínicos se tomó de evidencia de la vida real a partir de la información de cada una de las unidades de servicios de salud de Compensar EPS en Bogotá. Se reunieron los resultados obtenidos en los exámenes (prueba de HPV, citología refleja y colposcopia-biopsia) de la cohorte de mujeres que asistieron a su jornada de tamizaje de cáncer de cuello uterino. La información de los resultados de la citología convencional se obtuvo de otra unidad de servicios de salud que ha utilizado esta estrategia de tamizaje. Para ambos casos, se buscó mantener constantes el total de mujeres analizadas, el rango de edad, las características socioeconómicas y el tiempo de implementación de la estrategia de tamizaje (2015 a 2018) (cuadro 1). No fue posible calcular la especificidad de las pruebas diagnósticas debido a que las mujeres con resultados negativos no se someten a prueba confirmatoria.

**Cuadro 1:** Prevalencia resultados clínicos

Parámetro	%	Referencia
% cohorte con resultado ASC-US	9,04	Cohorte Compensar USS de tamizaje con CCU
% cohorte con resultado LSIL	1,89	
% cohorte con resultado HSIL	0,14	
Prevalencia de VPH-AR	11,92	Cohorte Compensar USS de tamizaje con prueba de VPH y genotipificación
Prevalencia de VPH-AR 16 y 18	2,79	
Prevalencia de NIC 1	1,94	
Prevalencia de NIC 2	0,23	
Prevalencia de NIC 3	0,17	
Prevalencia de cáncer invasivo de cuello uterino	0,02	

VPH: virus de papiloma humano; VPH-AR: virus de papiloma humano de alto riesgo; ASC-US: células escamosas atípicas de significación indeterminada; HSIL: lesión intraepitelial escamosa de alto grado; LSIL: lesión intraepitelial escamosa de bajo grado; NIC: neoplasia intraepitelial de cuello uterino

Toda esta información se consolidó en un único documento de Microsoft Excel® de forma anonimizada y con el dato del resultado de la prueba de tamizaje de HPV. Así, el análisis se hizo a partir de una fuente secundaria de información extraída del registro histórico de datos de Compensar EPS, y no representó un riesgo al no contemplar intervenciones o modificaciones intencionadas de las mujeres que participaron por decisión propia en la toma de muestra para la detección de cáncer de cuello uterino. Asimismo, el horizonte temporal del estudio fue retrospectivo, ya que las estimaciones y posteriores análisis partieron de información recolectada en el marco de la práctica clínica habitual de Compensar EPS.

**Figura 3:**
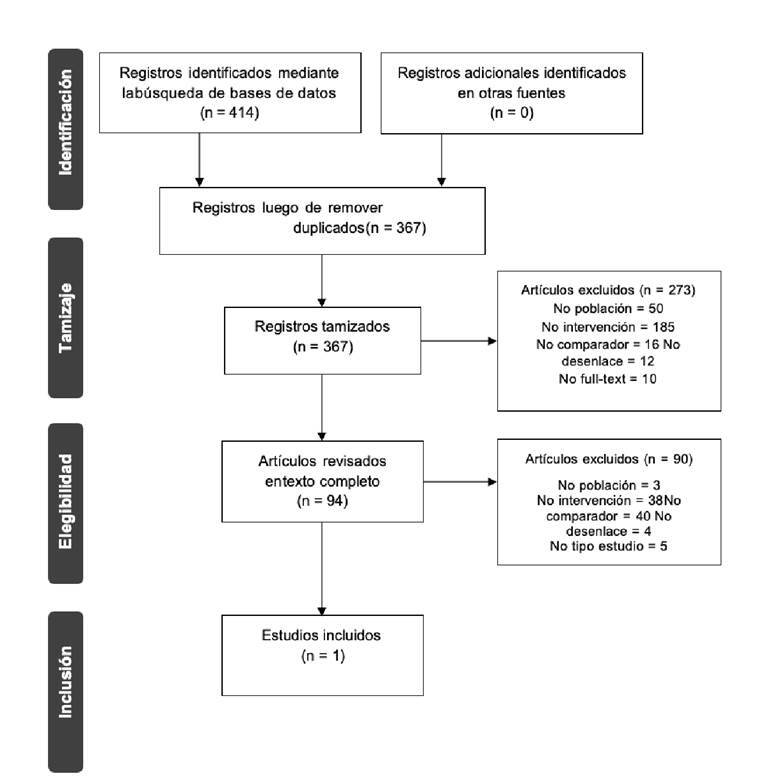
Diagrama de flujo de la búsqueda, tamizaje y selección de literatura

En aquellos casos en los cuales no se logró extraer información de la cohorte evaluada en cada unidad de servicios de salud, se optó por utilizar lo encontrado en la búsqueda estructurada de la literatura previa al estudio, en la cual se consideraron revisiones sistemáticas, metaanálisis, estudios de prueba diagnóstica y estudios observacionales. Los idiomas de las publicaciones fueron el inglés y el español, y debían estar disponibles como publicación completa publicada, en prensa o como literatura gris. La búsqueda se hizo en Medline (plataforma Ovid), EMBASE (plataforma Ovid), LILACS (Biblioteca Virtual en Salud-BVS, interfaz iAHx), Cochrane Central Register of Controlled Trials-CENTRAL (plataforma Ovid), y WHO International Clinical Trials Registry Platform-ICTRP. Los resultados se descargaron con un programa para el manejo de referencias y, posteriormente, se descargaron en Microsoft Excel® para seleccionar finalmente los estudios, como se observa en la figura 3.

La principal fuente de datos clínicos fue el estudio *Addressing the Need for Advanced HPV Diagnostics Study* - ATHENA, un estudio prospectivo multicéntrico con cerca de 47.208 mujeres mayores de 21 años que se sometieron a las pruebas de detección de cáncer de cuello uterino durante tres años para evaluar el valor de las pruebas Cobas™ ^14^. La colposcopia y la biopsia se consideraron las pruebas de referencia, por lo que se asumió que su sensibilidad y especificidad correspondían al 100 %.

*Datos de los costos*. El modelo incluyó los costos por paciente para el tamizaje, el diagnóstico de la enfermedad y el tratamiento clínico en aquellos casos con resultados confirmatorios para neoplasia intraepitelial de cuello uterino (grado 2+) y cáncer invasivo de cuello uterino. Todos los costos fueron reportados por la EPS Compensar y los de los tratamientos corresponden al costo promedio anual reportado en el 2018 (cuadro 2). Los costos de detección se calcularon sumando los costos unitarios para las pruebas individuales con los de una visita al consultorio. Los costos de diagnóstico incluyeron la visita al consultorio para el seguimiento del diagnóstico, y los costos de la colposcopia y la biopsia. Los costos del tratamiento se estimaron en función de los promedios en todas las etapas y opciones de tratamiento contempladas por Compensar EPS. No se incluyeron los costos indirectos o de rehabilitación.

**Cuadro 2:** Características de los costos incluidos en el análisis

Costos del modelo	Intervención o procedimiento	Valor total (COP)
Costos del tamizaje		
Consulta de medicina general	Actividades de promoción y mantenimiento por curso de vida	16.570
Prueba de citología vaginal	Toma de citología vaginal convencional y seguimiento por enfermería de resultados positivos	13.310
Prueba de citología vaginal	Procesamiento de citología vaginal	9.250
Prueba de ADN del HPV	Toma, procesamiento, lectura y seguimiento por enfermería de la prueba de ADN del HPV	80.210
Costos del diagnóstico		
Colposcopia	Colposcopia	84.200
Biopsia	Biopsia más lectura básica	44.360
Vigilancia de NIC2	Visita de enfermería (seguimiento del diagnóstico)	10.737
Costos del tratamiento		
Tratamiento para la NIC2	Tratamiento para la NIC (grado 2+) incluidas las consultas de gineco- oncología, conización y crioterapia	1’400.000
Tratamiento para el CCI	Tratamiento para el cáncer invasivo de cuello uterino	12’238.597

Fuente: Compensar EPS

Todos los precios son en pesos colombianos.

*Análisis de sensibilidad*. Se hizo un análisis de sensibilidad determinístico de una vía para estimar cómo el cambio en cada variable (clínicas y de costos) podía incidir en los resultados del impacto presupuestal. Las entradas clínicas asumieron una distribución beta, en tanto que, las de los costos, una distribución gamma en la que cada una de las entradas varió ±20 %.

## Resultados

### Impacto clínico

**Figura 4:**
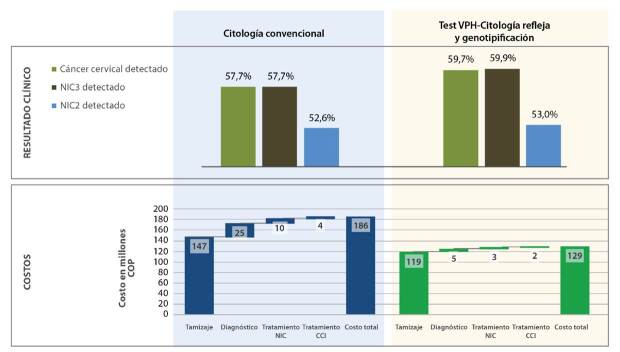
Resultados clínicos y costos

El impacto de la estrategia de detección en la enfermedad fue mejor en el escenario de la prueba de HPV, con una estimación de la incidencia anual de cáncer en la cohorte tamizada de 6,20 por 100.000 mujeres y de 0,73 por 100.000 mujeres en el caso de la citología. En la figura 4, se observan estos resultados y el porcentaje de detección de NIC2 y NIC3.

### Impacto financiero

Buscando simular el escenario más cercano a la realidad posible, se determinó el total de mujeres que en el seguimiento de la cohorte de la unidad de servicios de salud evaluada tuvo el segundo ciclo de la prueba de tamizaje antes de los tiempos propuestos en el planteamiento del modelo. Un total de 121 pruebas adicionales se hicieron a mujeres con resultado negativo, quienes repitieron la prueba a los 16 meses, aproximadamente, obteniendo nuevamente un resultado negativo. Esto puede deberse a la práctica habitual del esquema con citología, ya sea por solicitud médica o por el seguimiento de rutina autónomo de la mujer. El costo promedio de cada ciclo de tamizaje con la prueba HPV, la genotipificación y la citología refleja, se estimó en COP $129’201.363 frente a COP $183’309.952 con la citología convencional. El impacto presupuestal fue de COP $57’108.589 (30,7 %), lo que refleja menores costos, en promedio, por cada ciclo de tamizaje con la prueba de HPV.

En el modelo, los resultados tanto clínicos como de costos fueron muy sensibles al número de mujeres que asistió al segundo ciclo de tamizaje, lo que se debe a que este valor repercute en las estimaciones de progresión o regresión de la enfermedad de la cohorte inicial y, a su vez, en el uso de recursos y costos asociados con las pruebas de tamizaje, las consultas o los tratamientos que se requieran en esta segunda visita de rutina. Por ello, el valor de esta variable puede subestimar o sobreestimar el número de personas que abandona o que no cumple con sus jornadas de tamizaje en los tiempos propuestos. Sin embargo, esta estimación inferior o superior es la misma en ambos brazos del modelo y no afecta el valor diferencial del análisis de impacto presupuestal. El costo de ambas estrategias de tamizaje, en mayor medida la prueba de HPV, generó cambios en los resultados del caso de base, pero sin afectar la conclusión.

## Discusión

Durante más de 40 años, los programas de tamizaje de cáncer de cuello uterino en Colombia se han basado en la citología cervicovaginal convencional; sin embargo, el impacto en la mortalidad no ha sido tan representativo como en los países desarrollados ^2^. Por ello, se ha venido desarrollando un marco legal y normativo en torno al control del cáncer que busca, entre otras cosas, avanzar progresiva y gradualmente en la inclusión de la prueba de HPV como herramienta de tamizaje para el cáncer de cuello uterino, siguiendo la ruta de promoción y mantenimiento de la salud ^15^.

Los resultados obtenidos en el caso de base que aquí se analiza, sugieren que dicha intervención genera ahorros en los costos promedio de cada ciclo de tamizaje. Además, es más probable que las células precancerosas se identifiquen inmediatamente durante la primera visita de detección, reduciendo significativamente los posibles falsos negativos, lo que permitiría un diagnóstico y un tratamiento oportunos, así como una reducción en el número y la frecuencia de las visitas de seguimiento requeridas. Asimismo, el aumento de los intervalos de detección de rutina a cinco años que se conseguiría con la prueba de HPV disminuye el número de visitas y pruebas de tamizaje anuales (cuadro 3), lo que representa una diferencia de 19 % (COP $27’821.106) en los costos promedio de las actividades propias del tamizaje.

**Cuadro 3:** Resultados obtenidos para cada uno de los escenarios en cada ciclo

	Citología convencional		Prueba de VPH con citología refleja y genotopificación	
Costos anuales (COP$)	Ciclo 1	Ciclo 2	Ciclo 1	Ciclo 2
Tamizaje:	281’773.706	12’015.084	217’876.424	20’270.155
Consulta de medicina general	101’394.509	4’332.829	34’347.244	1’462.474
Prueba de citología vaginal	138’048.287	5’899.133	5.554.921	17.936
Prueba de ADN del HPV	42’330.910	1’783.122	177’974.259	18’789.745
Diagnóstico:	48’525.885	2’048.621	10’209.694	47.014
Consulta de medicina general	5’540.370	233.898	1’165.676	5.368
Colposcopia más biopsia	42’985.515	1’814.723	9’044.017	41.647
Tratamiento:	27’266.551	990.057	9’527.782	471.657
Tratamiento para NIC2	10’586.347	398.597	3’622.000	128.623
Tratamiento para NIC3	8’223.114	309.218	2’910.925	130.287
Tratamiento para el cáncer invasivo de cuello uterino	8’457.090	282.242	2’994.857	212.748
Costo total anual	357’566.143	15’053.762	237’613.900	20’788.827

En cuanto al impacto de los costos del diagnóstico, la prueba confirmatoria implica el mayor gasto en los ciclos de las dos opciones: el de la colposcopia y la biopsia en la opción con citología ascendió a COP $22’400.119 y con la prueba de HPV resultó en COP $4’542.832 (80 % menos), ya que, en el caso de las mujeres con resultado de células escamosas atípicas de significado indeterminado (ASC-US), la sensibilidad y la especificidad de la citología obligan a la realización de la prueba confirmatoria.

A pesar de que el costo de la prueba de tamizaje con HPV es mayor que el de la citología, el costo promedio del tratamiento del cáncer es mucho más elevado que el de cualquier método de detección oportuna ^16^. Es importante resaltar la importancia de considerar el cumplimiento de los ciclos de tamizaje, pues el ausentismo puede afectar la probabilidad de éxito en la detección de lesiones precancerosas y la necesidad de hacer diagnósticos en estadios avanzados. Esto quedó evidenciado en un estudio en que se concluyó que la falta de seguimiento puede aportar el 13 % de los registros de cáncer de cuello uterino invasivo ^17^.

Por otra parte, clasificar la infección del HPV e identificar los genotipos de mayor riesgo oncogénico proporciona información crítica para fortalecer la gestión del manejo adecuado de las pacientes. El horizonte temporal de seguimiento de la cohorte es una de las principales limitaciones del estudio, pues se extendió hasta tres años y solo en aquellas mujeres que siguieron afiliadas a la misma aseguradora y participaron de la jornada de tamizaje en la misma unidad de servicios de salud. A pesar de que una lesión preneoplásica de cuello uterino puede progresar a cáncer de cuello uterino en menos de 12 meses, por lo general toma varios años, por lo que un seguimiento más prolongado de las cohortes permitiría recolectar aún más información.

El modelo de base del análisis del impacto presupuestal se ha adaptado a diferentes contextos, tomando como dato inicial el del estudio ATHENA y la información de cohortes reportada en la literatura científica. Se encontraron reportes de la simulación con el mismo modelo en Alemania, Estados Unidos, Alemania y Bélgica, en los que las principales diferencias han radicado en los escenarios de los esquemas planteados, el aumento del rango de edad de las mujeres y algunos ajustes relacionados con el contexto específico de cada país, pero en todos los casos, la estrategia de tamizaje con la prueba de HPV resultó en el ahorro de costos comparada con la citología, principalmente por el aumento en los intervalos de detección de rutina de tres a cinco años ^13^^,^^17^^,^^18^^,^^19^.

En Colombia, Gamboa, *et al.,* evaluaron en el 2008 la costo-efectividad de la prueba HPV frente a la citología convencional y la ausencia de tamizaje utilizando un modelo de Markov basado en la historia natural del cáncer de cuello uterino. Los resultados evaluados fueron la mortalidad, los años de vida saludables (AVS) y los costos directos asociados. Los autores concluyeron que el tamizaje con la prueba de ADN-HPV era costo-efectiva para Colombia, dando un valor de la razón de costo-efectividad incremental (ICER) de USD $44/AVS (dólares por año de vida saludable) y costos de la prueba menores de USD $31 ^19^.

En conclusión, el tamizaje con la prueba de HPV resultó en el ahorro de costos en comparación con la citología convencional en mujeres de 30 a 65 años de edad atendidas en una institución prestadora de servicios de salud colombiana. La estrategia de intervención ha demostrado tener un mayor impacto en la detección primaria de mujeres en riesgo y lograría ahorros significativos, evitando costos innecesarios en pruebas confirmatorias. Esta información contribuye a la toma de decisiones en torno al mejor uso de los recursos disponibles. La implementación de la estrategia de vacunación generalizada y los programas de tamizaje primario de la infección de AR- HPV, contribuirían a una reducción significativa de las muertes por cáncer de cuello uterino.

## References

[1] The American Cancer Society Prueba de VPH ADN.

[2] Ministerio de Salud y Protección Social Guía de práctica clínica para la detección y manejo de lesiones precancerosas de cuello uterino. Guía para pacientes y cuidadores. Colombia 2014.

[3] Bray F, Ferlay J, Soerjomataram I, Siegel RL, Torre LA, Jemal A. (2018). Global cancer statistics 2018: GLOBOCAN estimates of incidence and mortality worldwide for 36 cancers in 185 countries. CA Cancer J Clin.

[4] Martínez-Gómez VM, Martínez JC. (2017). Protocolo de vigilancia en salud pública de cáncer de mama y cuello uterino.

[5] Saslow D, Solomon D, Lawson HW, Killackey M, Kulasingam SL, Cain J (2012). American Cancer Society, American Society for Colposcopy and Cervical Pathology, and American Society for Clinical Pathology screening guidelines for the prevention and early detection of cervical cancer. CA Cancer J Clin.

[6] Cuenta de Alto Costo (2016). Indicadores prioritarios para la medición, evaluación y monitoreo de la gestión de riesgo por parte de aseguradores y prestadores en pacientes con cáncer de mama y cuello uterino en Colombia.

[7] Rincón-Martínez LM, García-Peralta DM. (2012). Las pruebas de ADN para el virus papiloma humano -VPH-. Hechos & Acciones.

[8] Heideman DAM, Hesselink AT, Berkhof J, van Kemenade F, Melchers WJG, Fransen-Daalmeijer N (2011). Clinical validation of the cobas 4800 HPV test for cervical screening purposes. J Clin Microbiol.

[9] Roche Molecular Systems Inc Prueba cobas® VPH. Cobas®. 2016.

[10] Instituto Nacional de Cancerología (2007). Recomendaciones para la tamización de neoplasias del cuello uterino en mujeres sin antecedentes de patología cervical (preinvasora o invasora) en Colombia.

[11] Rincón-Martínez LM, García-Peralta DM. (2012). Las pruebas de ADN para el virus papiloma humano -VPH-. Los nuevos retos en la tamización para cáncer de cuello uterino.

[12] Wright TC, Stoller MH, Behrens CM, Apple R, Darion T, Wright TL. (2012). The ATHENA human papillomavirus study: Design, methods, and baseline results. Am J Obstet Gynecol.

[13] Ministerio de Salud y Protección Social (2018). Resolución No. 3280 de 2018. Por medio de la cual se adoptan los lineamientos técnicos y operativos de la ruta integral de atención para la promoción y mantenimiento de la salud y la ruta integral de atención en salud para la población materno perinatal y se establecen las directrices para su operación. Minsalud.

[14] Ministerio de Salud y Protección Social (2012). Lineamientos técnicos y operativos para la vacunación contra el virus del papiloma humano (VPH). Guía de práctica clínica para la detección y manejo de lesiones precancerosas de cuello uterino. Guía para pacientes y cuidadores.

[15] Colín MC, Domínguez MV, Mendieta H, Rojas IJ, Romero MS., Ramírez N, Domínguez MV (2017). Temas selectos de biomedicina en ciencias de la salud.

[16] Wright T, Huang J, Baker E, Garfield S, Hertz D, Cox JT. (2016). The budget impact of cervical cancer screening using HPV primary screening. Am J Manag Care.

[17] Karl A, Petry U, Barth C. (2017). A model to evaluate the costs and clinical effectiveness of human papilloma virus screening compared with annual papanicolaou cytology in Germany. Eur J Obstet Gynecol.

[18] Tjalma WAA, Kim E, Vandeweyer K. (2017). The impact on women’s health and the cervical cancerscreening budget of primary HPV screening with dual-stain cytology triage in Belgium. Eur J Obstet Gynecol.

[19] Gamboa O, Chicaíza L, García M, Díaz J, González M, Murillo R (2008). Cost-effectiveness of conventional cytology and HPV DNA testing for cervical cancer screening in Colombia. Salud Pública Méx.

